# On an Epidemic Skin Disease

**Published:** 1893-06

**Authors:** 


*On an Epidemic Skin Disease.* By THOMAS D. SAVILL, M.D. Pp. 64. London: H. K. LEWIS. 1892.

We have perused Dr. Savill's book with great interest and great care, because it is an elaborate and well-written record of an outbreak of a skin affection which, in our present knowledge, is, to say the least, uncommon, although we have reason to believe that cases in many respects very similar have occurred elsewhere than in certain medical institutions in the west part of London, during the year 1891. Dr. Savill gives us a record of 163 cases of this disease which came under his observation at the Paddington Infirmary during the summer and autumn of that year. Then 193 cases occurred at the Marylebone Infirmary, 4 at St. Mary's Hospital, 38, at least, at the Hanwell Asylum, and 25 at the Lambeth Infirmary. The death-rate of Dr. Savill's cases was as high as 12.8 per cent., and was much higher in males and the aged than in females.

The year 1891, possibly from meteorological causes, was remarkable for a good many unusual manifestations of disease. It was in that year that the cases of rötheln occurred at Clifton

College which were very graphically recorded by Dr. Fyffe in the *Bristol Medico-Chirurgical Journal* for March, 1892.

As regards the London cases, we do not consider they were either eczema or pityriasis rubra, although there can be no question that eczema preceded, or was associated with, the disease, and that many of them, in their exfoliative processes, resembled pityriasis rubra. However, at present it is better not to go beyond applying an appellation to the group or rather groups of symptoms described by Dr. Savill, and there seems no reason to object to the one adopted by him; viz., "General Exfoliative Epidemic Dermatitis."

